# A qualitative study of continence service provision for people living with dementia at home in the UK: Still inadequate?

**DOI:** 10.1371/journal.pone.0268900

**Published:** 2022-05-26

**Authors:** Cathy Murphy, Christine De Laine, Margaret Macaulay, Mandy Fader

**Affiliations:** School of Health Sciences, University of Southampton, Southampton, United Kingdom; University of Alberta, CANADA

## Abstract

**Introduction:**

Incontinence is a major problem for people with dementia (PWD) and their family/friend caregivers, often causing substantial harm, including residential care admission. The incontinence needs of PWD are complex and different from those of people without dementia.

The aim of this study was to investigate carer and nurse perceptions of continence service provision and potential improvements.

**Methods:**

A secondary analysis of qualitative data was undertaken. Semi-structured interviews (n = 45) were undertaken with PWD, family caregivers and healthcare professionals (continence or dementia nurses) in the UK. PWD and caregivers were recruited via www.joindementiaresearch.nihr.ac.uk and via dementia/carer groups. Nurses were recruited via their employers. Framework analysis was used. The COREQ Research guideline statement assists reporting.

**Results:**

Four themes were found. Firstly, there was a lack of awareness of the service and waiting time. Many caregivers were unaware of continence services and dementia nurses often viewed it as a pad provision service. Caregivers reported long waits not meeting their urgent needs. Secondly, product provision was often inadequate. Most caregivers self-purchased all or many products and substantial variation in product provision was found. The number of products provided was often inadequate. Thirdly, a sense that “nothing can be done” was observed by some nurses and caregivers. Caregivers believed that, if nothing else, care information should be provided. Finally, suggestions for improvements were made, including proactive service signposting, joint clinics with dementia services, improved information before crisis point, dementia training for continence nurses and improved product provision.

**Conclusion:**

Continence service inadequacies for PWD and caregivers have been reported for many years. This study demonstrates service provision remains unsatisfactory in the UK. Stakeholders propose a range of service improvements. It highlights that listening to the voices of PWD, caregivers and nurses is crucial for services seeking to improve continence services for PWD living at home.

## Introduction

The majority of people with dementia (PWD) live at home, and most are supported in their day-to-day activities by one or more family or friend unpaid caregivers [1]. This population is at considerably higher risk of developing incontinence than people without dementia of the same age [2, 3]. Incontinence often causes substantial harm to health and well-being [4] and can additionally lead to a breakdown in care at home resulting in admission into residential care [5]. In the UK, the inadequacy of the support received by PWD living at home with incontinence and their family caregivers was reported a decade ago [6]. This paper explores whether any progress has been made.

Due to the layering of physical and cognitive factors, the incontinence needs of PWD are complex and multi-faceted with different characteristics to those of people without cognitive impairment [4, 7]. It is not just the PWD who is affected by the incontinence; their caregivers often also face negative consequences. Over 670,000 unpaid caregivers for PWD provide support worth an estimated £11 billion per annum in the UK alone [1], but many can become overwhelmed by the workload [6] and incontinence (urinary or bowel) has been identified as a risk factor for permanent admission into residential care [8].

Despite this, there is a lack of evidence for interventions to support this population and their caregivers [9, 10]. Furthermore, current UK based clinical or commissioning guidance, such as National Health Service (NHS) England Excellence in Continence Care [11], Minimum Standards for Continence Care in the United Kingdom [12] or Royal College of Nursing bowel care guidance [13] neglect to address this population’s disparate needs. This is not an issue that is specific to the UK; the lack of guidance has been noted internationally [14]. Therefore, it is perhaps to be expected that individual community continence services (within the UK and elsewhere) do not commonly have policy with content specifically aimed at meeting the needs of PWD [14, 15].

Given the lack of dementia related policy or guidance, it is unsurprising that the support that PWD and their caregivers have received to cope with incontinence at home has been reported as unsatisfactory. In 2011, a seminal paper reported that continence services failed to meet the needs of caregivers of PWD, stating that services could be more proactive and open in enquiring about problems, that clinicians needed enhanced dementia skills and that services in the UK were inappropriate and unresponsive [6]. This paper seeks to establish whether any improvement has been achieved in the last decade and achieves its specific aim of investigating carer and nurse perceptions of the current continence service provision and potential for improvement. This study took place in England where community-based continence services are commissioned to provide comprehensive continence assessment, treatment and management services to their local population.

## Methods

Secondary analysis of a qualitative study on the causes, consequences and potential solutions of incontinence and dementia at home [4] was undertaken to address the research aim. PWD, caregivers and nurses (continence or dementia, registered or non-registered nurses) took part in semi-structured interviews with CM (post-doctoral researcher experienced at undertaking qualitative interviews and registered nurse). The research question being addressed by the secondary analysis reported in this paper was derived from the results of the primary analysis which indicated that the interview data contained rich findings that warranted more detailed exploration of views on continence services. PWD and caregivers were recruited via www.joindementiaresearch.nihr.ac.uk and dementia/carer groups. Nurses were recruited via their employers (two NHS Community Trusts and Dementia UK, representing four continence services). A purposive sampling approach was used to gather data from a broad range of participants (by sex, PWD/carer relationship and living arrangements for PWD/carer participants and professional background for healthcare professional (HCP) participants). All participants were provided with information sheets explaining the research, including the purpose. For PWD, a Process Consent approach was used to facilitate ethical and safe inclusion of participants [16]. Interviews took place in private spaces in homes or workplaces using pilot-tested topic guides; field notes were taken as aides memoire. Interviews were digitally recorded and transcribed verbatim. To protect confidentiality, all data was de-identified and unique participant codes used. A framework approach was taken for this secondary analysis with the goal of addressing the research aim, following the steps of Familiarisation, Constructing a thematic Framework, Indexing and sorting, Data summary and display and Mapping [17] with CM and CdL undertaking coding using Microsoft Word software. Framework analysis sits under the umbrella of thematic analysis [18] and is suited to studies such as the one reported here with goals pre-determined by applied research objectives, for example to inform policy development [19]. Rigour was maintained through the use of strategies such as memos and audit trails. A reflexive approach was taken to actively identify any research team biases. This approach has been recognised as important in maintaining the credibility of qualitative research [19]. A source of potential bias identified was that the research team had professional backgrounds in continence nursing and research and as such took steps, such as deviant case analysis, to counteract any impact of pre-conceived beliefs.

The Consolidated Criteria for Reporting Qualitative (COREQ) Research guideline statement assists the reporting [20] (checklist provided in S1 File). Ethical Approval was received from NHS Health Research Authority.

## Results

45 people (26 family caregivers, two people with dementia, nine continence nurses and eight dementia nurses) took part. The PWD and caregivers came from a geographical area covering seven NHS community trusts (each with their own continence service) in the south of England, including both rural areas and cities. A summary of participants including relationship with the PWD is given in Table 1. Four main themes were derived from the data and are explained with illustrative quotes below.

**Table 1:** Summary of participants.

Group	Sex	Details
**PLWD**		**Relationship with carer**
PLWD 1	F	Wife of current carer (H6)
PLWD 2	M	Husband of current carer (W10)
**Carer**		**Relationship with PLWD**
W1	F	Wife to husband (former carer)
W2	F	Wife to husband (former carer)
W3	F	Wife to husband (current carer)
W4	F	Wife to husband (current carer)
W5	F	Wife to husband (current carer)
W6	F	Wife to husband (former carer)
W7	F	Wife to husband (former carer)
W8	F	Wife to husband (former carer)
W9	F	Wife to husband (former carer)
W10	F	Wife to husband (current carer)
H1	M	Husband to wife (current carer)
H2	M	Husband to wife (current carer)
H3	M	Husband to wife (current carer)
H4	M	Husband to wife (current carer)
H5	M	Husband to wife (former carer)
H6	M	Husband to wife (current carer)
H7	M	Husband to wife (current carer)
S1	M	Son to father (former carer)
S2	M	Son to mother (current carer)
Sil1	M	Son-in-law to mother-in-law (current carer)
N1	F	Niece to aunt (former carer)
D1	F	Daughter to mother (current carer)
D2	F	Daughter to mother (former carer)
D3	F	Daughter to mother (former carer)
D4	F	Daughter to father (former carer)
D5	F	Daughter to mother (former carer)
Dil1	F	Daughter-in-law to mother-in-law (former carer)
**Nurse**		**Specialism, registered/non-registered (registered refers to a qualified nurse who is registered with the Nursing and Midwifery Council in the UK)**
ConN1	F	Continence, non-registered
ConN2	F	Continence, registered
ConN3	F	Continence, non-registered
ConN4	F	Continence, non-registered
ConN5	F	Continence, non-registered
ConN6	F	Continence, non-registered
ConN7	F	Continence, registered
ConN8	F	Continence, registered
ConN9	F	Continence, registered
DemN1	F	Dementia, registered
DemN2	F	Dementia, registered
DemN3	F	Dementia, registered
DemN4	F	Dementia, registered
DemN5	F	Dementia, non-registered
DemN6	F	Dementia, registered
DemN7	F	Dementia, registered
DemN8	F	Dementia, registered

### 1. Lack of awareness of the service & waiting time

A substantial minority of caregivers were either unaware of the existence of a continence service or were unsure what the service provided and whether it was relevant to their needs. As one daughter said, *“I didn’t know there was an incontinence clinic*. *That was a surprise”* (D1). This issue was seen as partly caused by a lack of communication between health and social care professionals (GPs, Dementia nurses or others) and caregivers: *“Continence is something that is swept under the carpet*. *There are so many people that have it but it’s not something that is considered to be a topic that you’d chat about in normality*. *So nurses*, *caregivers*, *people like that don’t really talk about it*.*”* (DiL1).

Some dementia care nurses saw the continence service merely as a pad provision service that could help PWD and caregivers save money: *“We do refer fairly often to the bladder and bowel service*. *I’d say that’s probably more for financial support than anything though*.*”* (DemN2). Dementia nurses were often unaware of any other service provided.

When caregivers did contact their local continence service, they often reported a long wait that did not meet their urgent needs: *“I did phone someone and it was going to be several weeks before they could do anything and I thought that’s a bit rubbish*, *you’d think there would be someone that even if you had to go somewhere they could give you advice that day*, *but oh no it doesn’t work like that*.*”* (H3). Several caregivers stated that they felt continence management was an urgent need and the length of time before their first appointment (often several months) left them trying to work out how to cope by themselves or to try to get support from other sources. *“Some people are really desperate*, *I mean I know somebody whose wife is now in a home but he was really desperate and they were just like that*. *Absolutely hopeless*.*”* (W5) In some cases, continence services sign-posted caregivers to other support sources, including product manufacturers: *“I phoned up the continence service to get the appointment and they told me that in the meantime because they wouldn’t be able to come out to me for a long time what I should do is phone up [pad brand name] and talk to them*.*”* (W4)

### 2. Product provision

Whilst a minority of caregivers reported that all of the products used by the person they cared for were provided by the continence service, the majority self-purchased some or all of the products they used. This was a tremendous source of frustration for many caregivers: *“He’s 98 for goodness sake*, *he’ll be 99 in February*. *I just think he fought for this country in the war*, *he’s never been on benefits*, *he’s always found himself work after he came out of the forces and retired*. *He’s never asked for anything and they won’t even give him continence products*.*”* (D4).

The main reasons for self-purchase were that either the person had not been referred to the continence service or the continence service did not provide the design, absorbency level or number of products that met the PWD’s needs. The most mentioned gap in provision was around disposable pull-on pads (also known as pull-ups). A minority of caregivers asking for this design received them in the quantity requested. However, the majority did not: *“I said are they [product being offered] the pull-on ones and she said no*, *no they’re just the wrap around*. *I said not interested*. *I never bothered to push it any further*. *Others I’ve spoken to through my contacts they’ve all said exactly the same*, *they only provide the wrap around certainly around here*.*”* (H3). A dementia nurse explained why many PWD cope better with pull-ons than other designs: *“Although the majority of people prefer the pull-up pads*, *I don’t think they supply them so that’s a bit of a gap really*. *The person with dementia likes to have the pull-ups because that’s like a pair of pants*, *it’s very dignified …*.*I mean the pull-up pants are the most normal thing that somebody with dementia would understand because all their life they’ve put a pair of pants on and pulled them up*. *They haven’t necessarily had something between their legs and stuck at the sides*.*”* (DemN3)

The number of products provided per day was generally limited to three or four, sometimes less for relatively expensive products such as pull-ons.: *“Now of course they issue you one per day of those things*, *but my mum might soil seven or eight in a day*. *And I tried to explain to them it would be much better if I could get something smaller*. *But they said no that’s what they prescribed and that was what she needed*.*”* (D2). Absorbency was also restricted by some providers. Whilst it is broadly accepted that continence services do not provide absorbent products for light urinary incontinence, some caregivers also reported that products for very heavy incontinence were also not available: “*There was funding for ones up to a certain absorbency but the ones that we needed that were greater absorbency we had to pay for those*.*”* (D5)

Along with the lack of products provided, there was often a lack of information on the range of designs available. Information was often limited to the product (or products) that the service was willing to provide without any explanation of other options available for self-purchase. Several caregivers explained that when they later found out about different product designs from other information sources (e.g. nursing homes, pharmacies or on-line) that they were frustrated with the lack of advice. This was particularly infuriating for caregivers whose spouse or family member had already been admitted into a nursing home when they learned about more appropriate designs as illustrated by this wife’s comments: *“And they don’t think how much easier it would have been for me to have those wrap around ones that I didn’t even know existed until we went to the [nursing] home*.*”* (W6).

### 3. Nothing can be done

A sense of therapeutic nihilism was expressed by some nurses and caregivers. Common curative treatments (such as pelvic floor exercises) were believed to be unlikely to work due the nature of dementia and little else was available beyond pads. Some dementia nurses commonly did not discuss incontinence (“*They just seemed to say oh well it’s his dementia*. *In fact*, *I never had anybody advise me on his incontinence at all in any form*. *They never even referred to it*.*”* W6) or refer PWD to the continence service (except for pads) (*“Because my wife really couldn’t respond to any advice or instructions I think they didn’t think it was worthwhile [referral to continence service]”* H5).

Some caregivers agreed with many dementia nurses that, for PWD, the continence service was no more than a ‘pad service’: *“The continence service seems to be a grudging nurse filing in a form*, *not wishing to be rude about the nurses*, *because it’s not an exciting thing to do and they’ll fill it in at their convenience rather than necessarily at the convenience of the people that are coping with the consequences of incontinence*. *And then you will be prescribed a pad and that’s you done*.*”* (Dil1)

When asked about dementia related training, all continence nurses responded that they had either received none or very little: *“We attend the dementia training which is a one-day course which was cancelled when I booked it so I’ve never actually attended it*. *But we use the Dementia UK continence leaflet which we give out to be patients*.*”* (ConN9).

Several continence nurses explained that they assess and treat PWD in the same way as patients without dementia, but acknowledge that treatment strategies are less likely to work: *“If you’ve got two people sat there we wouldn’t treat them any different but Doris isn’t going to get as better as Florence because Florence is going to do everything we’ve told her*. *Caregivers can support and help but it’s that cognition*.*”* (ConN2).

However, several continence nurses expressed frustration with the restrictions placed on them and thought that the service could be improved: *“Just dishing out pads to people is not the solution*. *And it drives me insane*. *There are other things you can do*. *If we had the resources and the services and the people to go out there we could do so much good work without medicines or pills or anything else*, *just by supporting people and letting them know that’s OK*, *that it’s quite normal*.*”* (ConN8) Caregivers confirmed that, whilst accepting that it might be impossible to cure the incontinence, as a minimum they would like much more information and support on how to cope.

### 4. Suggestions for improvements to continence services

Participants were asked what could help improve continence services for PWD and their caregivers. Common suggestions were:

- Proactive signposting to the continence service from other care professionals at an early stage. As one carer said: *“Just an info sheet—once the incontinence starts it’s too late then*.*”* (W1). A continence nurse agreed with the need for early information: *“In an ideal world what you’d have is you’d have that support and that communication at that point of diagnosis before it gets to the point where they’re pulling their hair out*.*”*(ConN8)
- Practical information on what might happen and how to manage incontinence. Many caregivers thought more information would be valuable: *“I think it’s their responsibility to actually tell people*. *Here’s this leaflet*, *you might never need it*, *would be my sort of thing*, *you might never need it*.*”* (W5). Practical tips including a fact sheet on fundamental care would be welcomed by many as one husband explained: “*And information on general how to clean somebody*. *I know it’s a basic thing but most men or many men didn’t get involved with doing their children necessarily …*. *And they don’t know [what to look for]*. *They’re not a woman*, *what are the things you’ve got to be careful about*?*”* (H6). Several caregivers mentioned useful knowledge such as RADAR keys (keys that provide access to public toilets specifically allocated for those with disabilities in the UK): *“The disabled toilets which are the only ones with the right facilities you have to have a key for and I had no idea you have to have a key and so we were stuck*. *The first response from the continence people could just be to send you out a factsheet with issues”* (H5).
- Dementia & incontinence service framework. Many nurses and some caregivers highlighted the need for integrated services: “*I know everybody is different and it never goes the same way for everybody but there are certain points that everyone must reach*, *one being continence*, *it would be nice if there was some framework in place that*. *We’ve made it up as we go along*, *completely made it up*.*”* (H2). A joint clinic model sometimes used within other clinical areas (e.g. multiple sclerosis) was suggested by several nurses. The need for better training for nurses (predominantly dementia skills for continence nurses, but also continence knowledge for dementia nurses) was highlighted by nurses themselves (particularly continence nurses) and also caregivers: “*I was thinking to myself all these people are the experts*, *they know everything*, *but of course it’s only later that you realise that they are all disparate*.” (D2). Several participants (both caregivers and nurses) observed that seeing people in their own homes would be an important part of an effective service: *“We do feel that patients often need to be seen in their own home to see what the configuration is at home*, *what’s happening to make a better assessment*. *They might just need a commode by the bed*. *We can’t do that unfortunately*.*”* (ConN9).
- Improved product and product information provision (both quantity and range of designs and absorbencies). Together with improved information, this was the most commonly suggested improvement, particularly by caregivers. They wanted not just a wider selection of designs in adequate quantities, but also better information of the available options (even if not directly available from the continence service).

## Discussion

In 2011, in their influential work “A taboo within a stigma? a qualitative study of managing incontinence with people with dementia living at home” Drennan et al stated that “Professional responses can be poor, and services inadequate” [6]. This paper indicates that this situation does not appear to have improved over the last decade; services remain insufficient to meet the needs of PWD and their caregivers.

Help-seeking for incontinence has long been acknowledged as problematic [21, 22] and can be particularly difficult for caregivers seeking help for their family member or friend [6]. This is even more challenging when caregivers are not aware of the available services. Caregivers want to see more proactive signposting and information provision at an earlier stage to help avoid the process of trial and error and often crisis that many go through. Nurse participants suggested joint clinics held with nurse specialists of other patient populations (specifically, multiple sclerosis) and indicated that similar models (i.e., joint dementia and incontinence clinics) could be valuable for PWD. Where joint clinics are not available, proactive screening measures have proven effective in other clinical specialities (e.g., the regular use of short bladder management questionnaires at neurology clinics–[23]) and could have potential benefits if provided at dementia appointments. As a minimum, caregivers would like to receive practical guidance on coping with incontinence. A ‘Handbook’ to provide detailed practical incontinence and toilet-use guidance to caregivers of PWD is under development by the authors, but in the meantime, organisations including the Alzheimer’s Society (UK), Alzheimer’s Association (USA), the Continence Foundation of Australia and Alzheimer Society (Canada) provide useful care tips on their websites.

Once PWD and caregivers have found continence services, they want to be met with more comprehensive, holistic support aimed at their specific requirements. Frustration with the ‘nothing can be done’ view of continence care was reported by caregivers and also by many nurses, particularly continence nurses who were frustrated by the lack of provision for this population. There appears to be a disconnect between what continence services offer (treatment to improve the level of incontinence leakage) and what caregivers want (more information and support with coping with existing incontinence). As Burholt and colleagues note, nurses internationally are hampered by the lack of evidence-based interventions or even expert based guidance on how to support PWD and caregivers and this contributes to the nihilistic approach [14]. Remarkably, international continence guidance “boldly suggests that ‘most people with a dementia diagnosis living in the community can potentially be managed in a similar way to any other community‐dwelling adults in line with current guidelines’ [24]. However, there is limited evidence to back up this assertion.” [14]. It is clear that dementia focused continence interventions to support both PWD and caregivers are urgently needed to inform policy and practice globally.

Product provision has long been a source of frustration for PWD and their caregivers, in the UK and elsewhere [6, 25] and it is known that inadequate product use can lead to harm for both the person and carer [26]. UK Clinical guidance states that products should be provided pending treatment, as an adjunct to on-going treatment and in the long-term if treatment is not successful [26]. A recent update of the Royal College of Nursing endorsed document, ‘Guidance for the provision of absorbent pads for adult incontinence–consensus document’ now includes the statement that two-piece continence products (where the pad is inserted into tight fitting underwear) might not be easily usable for some people, including those with dementia and wrap-around pads (also known as diapers) or pull-on pads should be offered. If consistently implemented, this guidance might help to alleviate some of the current provision disparity. A number of caregivers recalled learning about product designs that they felt could have helped them cope with the incontinence at home after the PWD had been admitted into a nursing home. This indicates that the adequate, properly informed provision of incontinence products has the potential to help avoid or delay nursing home admission in some cases.

The wide range of continence products available means that choosing the most appropriate designs can be challenging. The use of the evidence based, independent international website www.continenceproductadvisor.org can help people and their HCPs to choose the right products for individual needs. However, given the financial implications there needs to be transparency on the level of provision expected from community trusts. Analysis of the cost-effectiveness of optimal product provision might support the case for providing a wider range of designs in adequate quantities. However, this is not straightforward as the financial benefits of improved product provision are unlikely to sit within the same organisation as the fiscal burden; for example, in the UK the cost is with NHS community trusts, but the benefits are likely be for statutory social care providers (as well as individuals) due to the potential reduced need for domiciliary or residential care services. Patients bearing the financial burden of product purchase is not a situation unique to the UK; a study based in the UK, Germany, Poland and Spain found that 75% of people (not necessarily with dementia) covered least some of the cost [25] and it has been noted that Medicare in the USA covers the cost of urinary catheters, but not absorbent products [27].

Making progress on care for PWD is one of the long-term aims of the NHS and this paper describes service developments that caregivers and nurses agree would help improve incontinence care for PWD. Within England, it is now up to NHS England and individual Clinical Commissioning Groups (CCGs) to listen to the dissatisfaction expressed by these stakeholders, take note of their recommendations and consider ways to ensure better services. Researchers also need to work with stakeholders to develop evidence-based, innovative strategies to improve care. The recent development of the first internationally applicable Key Performance Indicators to measure the outcomes for the management of urinary and faecal incontinence based upon toileting and containment strategies [28] provides one example of how quality frameworks could be developed even prior to the development of evidence-based strategies.

The strength of this paper lies is in the multiple perspectives (carer, dementia nurse and continence nurse) it provides, but it does have some limitations. The key limitation is that despite considerable attempts at further recruitment, there was a lack of PWD participants. Secondly, the sample was entirely white British and does not capture a full diversity of views.

## Conclusion

Inadequacies in the provision of continence services for PWD and their caregivers have been reported for over a decade and this study demonstrates that service provision remains unsatisfactory in the UK. This opinion was expressed not only by caregivers, but also by both dementia and continence nurses, the majority of whom were frustrated by the service that they were able to provide. Considerably more work is required to commission services and develop effective interventions. The stakeholders participating in this study propose a range of service improvements. In addition to reiterating that the provision of continence support for PWD and their caregivers remains insufficient to meet needs, this paper outlines areas for potential improvement. It highlights that listening to the voices of PWD, caregivers and nurses is crucial for services seeking to improve the commissioning and delivery of continence services for PWD living at home.

## Supporting information

S1 File (PDF)
